# An Assessment and Comparison of the Efficacy of Two Licensed Tetravalent Leptospira Vaccines for Dogs Using an Improved Challenge Model

**DOI:** 10.3390/vaccines10091472

**Published:** 2022-09-05

**Authors:** Henricus Leo Bernardus Maria Klaasen, Mark van der Veen, Christina Maria Dorrestein-Spierenburg, Qi Cao

**Affiliations:** 1 Companion Animals Research and Development, MSD Animal Health, P.O. Box 31, 5830 AA Boxmeer, The Netherlands; 2 Clinical Research, MSD Animal Health, P.O. Box 31, 5830 AA Boxmeer, The Netherlands

**Keywords:** Leptospira, dog, vaccine, urinalysis, nephritis, Australis, Canicola, Grippotyphosa, Icterohaemorrhagiae

## Abstract

An objective of the present studies was to compare the protective effects of two European licensed canine tetravalent leptospirosis vaccines, Nobivac L4 and Versican Plus L4. Four similar challenge studies in beagle dogs were performed. In each study the dogs were assigned to one of three treatment groups as follows: group 1, Nobivac L4; group 2, Versican Plus L4; group 3, non-vaccinated control group. Two vaccinations were followed by a Leptospira challenge. Strains of the following serogroups were used for challenge: study 1, Grippotyphosa; study 2, Icterohaemorrhagiae; study 3, Canicola; study 4, Australis. Parameters of efficacy were antibody titres; body temperature; clinical signs; cultures of Leptospira bacteria from the blood, urine, kidney and liver; rapid urinalysis; macroscopic and histopathological examination at necropsy. It was concluded that compared to vaccination with Versican Plus L4, vaccination with Nobivac L4 resulted in generally better control of leptospirosis disease parameters after the challenge including a complete prevention of the clinical signs following a Grippotyphosa and Icterohaemorrhagiae challenge. In contrast, vaccination with Versican Plus L4 only prevented infection by Australis and shedding by Grippotyphosa and Australis but it did not lead to any statistically significant reduction of either infection or shedding following an Icterohaemorrhagiae challenge.

## 1. Introduction

Leptospirosis has been demonstrated in virtually all mammalian species and is almost certainly the most widespread global zoonosis, causing a worldwide public health and veterinary problem [1,2]. This disease is caused by infection with one of more than 230 serovars belonging to at least ten pathogenic species of the *Leptospira* genus [3]. The central factor in the epidemiology of leptospirosis is the renal carrier animal excreting leptospires into the environment [4], p100. Dogs are highly susceptible to infection and can be used as a sentinel species for the environmental risk to humans. In addition, while most human cases of leptospirosis are contracted from farm animals or rodents, the potential for infection from dogs should always be borne in mind [5].

Seroprevalence studies have suggested that the predominant and most widespread serogroups in dogs are Canicola, Icterohaemorrhagiae, Australis, and Grippotyphosa, with, additionally, Pomona being relevant in the USA and Hebdomadis in Japan [4], (p111), and [6]. A range of vaccines against canine leptospirosis have been licensed in Europe, the oldest of which being bivalent vaccines containing serovars Canicola and either Icterohaemorrhagiae or Copenhageni. More recently, trivalent and tetravalent vaccines have been introduced, containing either serovars of serogroups Grippotyphosa and Australis [7,8] or Grippotyphosa alone [9]. Efficacy claims for these licensed vaccines, described in the Summary of Product Characteristics (SPC) and the package insert text of each product, may include the reduction or prevention of infection (bacteraemia) and/or urinary shedding and/or clinical signs. The two tetravalent vaccines on the European market are Nobivac^®^ L4 (MSD Animal Health) and Versican Plus^®^ L4 (Zoetis): the former was licensed with claims of the reduction or prevention of infection and urinary shedding, while the latter was licensed with additional claims of the prevention of clinical signs [10,11].

To imply that there are efficacy differences between different vaccines based on differences in their respective SPC claims is scientifically incorrect and potentially misleading. The reason for this is that the efficacy claims in the SPCs are the outcome of separate dog efficacy studies conducted by different companies, with differences in study set-up, source and status of dogs, challenge strains, challenge models, etc. Only, a direct comparison of the efficacy of different vaccines in appropriate and valid studies involving both products can provide scientifically justified conclusions in this respect.

The main objective of the present studies was to assess the protective effects in dogs of the two tetravalent vaccines, Nobivac^®^ L4 and Versican Plus^®^ L4, in comparison with a non-vaccinated control group. The latter vaccine was used as a “positive control” for the protection from clinical signs of leptospirosis because in a prior publication, the absence of clinical signs in all vaccinates in each challenge study was described [8]. A secondary objective was to compare the efficacy of the two vaccines.

## 2. Materials and Methods

### 2.1. Experimental Design

Each study was divided into two parts (vaccination and challenge) which were performed at two geographically separated research facilities, which are further detailed under the Animals and housing section. Four similar studies in beagle dogs were performed. In each study, twenty-four puppies were randomly divided between the following treatment (vaccination) groups (8 dogs per group): group 1, Nobivac^®^ L4; group 2, Versican Plus^®^ L4; group 3, non-vaccinated control group. All groups were matched for gender and age (approximately 50% males and 50% females per group and approximately 6 weeks old at the start of the study). According to the manufacturers’ instructions for both L4 vaccines, the vaccination schedule consisted of two subcutaneous vaccinations, four weeks apart. The first vaccination was given at the age of six weeks and the second one at the age of ten weeks. The Leptospira challenge was performed three weeks after the second vaccination. Strains of the following serogroups were used for challenge: study 1, Grippotyphosa; study 2, Icterohaemorrhagiae; study 3, Canicola; study 4, Australis. In studies 1, 2, and 4 the challenge material was prepared from livers of infected hamsters. For further details of vaccinations and challenge, see Table 1.

The following parameters/tests were used to measure vaccine immunity and efficacy: titres of agglutinating serum antibodies against the four vaccine serogroups, body temperature, laboratory-confirmed (lab-confirmed) clinical signs, culture of challenge organisms from blood, urine, kidney and liver, rapid urinalysis (for details, see section under Rapid urinalysis), and macroscopic and histopathological examination at necropsy. Post-mortem examination of all surviving dogs was performed four weeks post-challenge.

In order to prevent clinical signs unrelated to the Leptospira challenge from confounding the true clinical effect of the challenge, only lab-confirmed clinical signs were included in the evaluation, which is analogous to how canine leptospirosis is diagnosed in a clinical setting. This meant that positive cases were those which, in addition to one or more suspect clinical signs, had at least one positive laboratory result/finding from the following: culture (of blood, urine, kidney or liver) for leptospires, urinalysis (for proteinuria or bilirubinuria), or typical pathology associated with leptospirosis (from macroscopic and histopathologic examination of various organs and tissues post-mortem).

### 2.2. Vaccines and Blinding

The vaccines were sourced from the following manufacturers: Nobivac^®^ L4, MSD Animal Health, Boxmeer, The Netherlands; Versican^®^ Plus L4, Zoetis, Louvain-la-Neuve, Belgium. In studies 1 and 2 the following batches of vaccines were used: Nobivac^®^ L4, batch 172668.1 and Versican Plus^®^ L4, lot 316222A02. In studies 3 and 4 Nobivac^®^ L4 batch 172714 and Versican Plus^®^ L4 lot 696223A01 were used. All vaccine batches used complied with the potency requirements of Monograph 0447 of the European Pharmacopoeia. In addition, all vaccine batches used were well within their expiry dates. Nobivac^®^ L4 is a non-adjuvanted vaccine prepared from inactivated antigens of serogroups Canicola, Icterohaemorrhagiae, Grippotyphosa, and Australis. Versican Plus^®^ L4 is an adjuvanted vaccine prepared from inactivated antigens of the same four serogroups and with aluminium hydroxide as the adjuvant.

The biotechnicians/animal caretakers, veterinarians, and pathologists of each research facility were blinded to the identity of the vaccines. The vaccines were blinded to the staff that performed the vaccination phase of the study (see Animals and housing) by being labelled as “vaccine A” and “vaccine B”. For blinding of the treatment groups, see sections: Vaccination and Challenge.

### 2.3. Animals and Housing, Randomisation and Blinding, and Ethical Review

Ninety-six conventional six-week-old male and female beagle pups, with no or very low titres of agglutinating serum antibodies against serogroups Canicola, Icterohaemorrhagiae, Grippotyphosa, and Australis, were purchased from Marshall BioResources (formerly Envigo) in Gannat, France. The vaccination phase of each study was performed in the dog facilities by the staff of Marshall BioResources. Approximately one week after the second vaccination, the dogs were transported to the dog facilities of MSD Animal Health in Boxmeer, the Netherlands for the second phase of the study in which the challenge and post-challenge sampling and monitoring were performed. After the transport, there was an acclimatisation period of two weeks prior to challenge. In the animal facilities of both study phases, the dogs were fed with commercial dog pellets and had free access to water. For individual identification of the dogs, subcutaneous chips (transponders) were used.

Prior to the start of each study at Marshall BioResources, the pups were randomised into three groups (*n* = 8 per group) using litter and sex as randomisation factors. In three of the studies, one dog had to be excluded prior to the challenge phase of the study due to health problems that were unrelated to the vaccinations. One of these dogs was a non-vaccinated control dog.

In both study phases (vaccination in France and challenge in the Netherlands), the staff performing clinical assessments and/or laboratory analyses were blinded to the group allocation. In the study protocol and the treatment and recording forms, blinded codes for the vaccines and vaccinated groups were used. In addition, for the challenge phase of each study, the animals of each treatment group were spread over various animal rooms.

All housing systems used in these studies fully complied with the requirements of the Federation of European Laboratory Animal Science Associations (FELASA). The studies described here were conducted after prior written approval by the responsible ethics review committees in France and the Netherlands, respectively. Thus, this work follows international, national, and institutional guidelines for humane animal treatment and complies with relevant legislation.

### 2.4. Vaccination

For age, time interval, administration route, dose, and injection site of the first and second vaccinations, see Table 1.

### 2.5. Challenge

Each study was carried out with a challenge strain of one specific serogroup and serovar of Leptospira. The following strains were used for the challenge: study 1, serogroup Grippotyphosa serovar Bananal/Liangguang strain 11808; study 2, serogroup Icterohaemorrhagiae serovar Copenhageni strain CF1; study 3, serogroup Canicola serovar Canicola strain Moulton; study 4, serogroup Australis serovar Bratislava strain LN0552-S/82/1409. Serovar identification of these strains was conducted by the OIE and National Collaborating Centre for Reference and Research on Leptospirosis (NRL) in Amsterdam, the Netherlands. Immediately prior to studies 1, 2, and 4, a hamster infection experiment was performed, as previously described [12]. Subsequently, dilutions of hamster liver homogenate were used to prepare the actual inoculum for the challenge of dogs. In study 3, a fresh in vitro culture of serovar Canicola strain, Moulton was used as challenge inoculum. The concentrations of Leptospira bacteria in the actual challenge inocula were as follows: study 1, 5.0 × 10^8^/mL; study 2, 7.0 × 10^7^/mL; study 3, 4.8 × 10^8^/mL; study 4, 3.5 × 10^7^/mL. For challenge routes and doses (ml per route of administration), see Table 1.

### 2.6. Serology

Blood samples from all pups were collected in plain serum tubes just before first vaccination, three weeks after the second vaccination (=pre-challenge), and one and four weeks post-challenge. Serum was obtained by centrifugation of the blood at 2000× *g* for 10 min. Titres of agglutinating serum antibodies against the four vaccine serogroups were determined using the microscopic agglutination test (MAT) according to standard procedures [13]. The live MAT antigens used belonged to the following serogroups and serovars: Canicola–Canicola, Icterohaemorrhagiae–Copenhageni, Grippotyphosa–Dadas, and Australis–Bratislava. Throughout all four studies, the titres of anti-Canicola agglutinating antibodies after two vaccinations were significantly lower in group 2 than they were in group 1. Therefore, in study 3 (with Canicola challenge), a growth inhibition test (GIT) was performed to find out whether neutralising antibodies against Canicola would show the same difference. The GIT was performed as described by De Nardi et al. [14], with some modifications. In short, sera were incubated after mixing with Leptospira cultures in Ellinghausen McCullough Johnson Harris (EMJH) medium containing 5-fluorouracil. Cultures without addition of sera were used as reference samples for normal growth (no inhibition). After incubation at 29 °C for 6 days, growth was assessed using dark field microscopy using the following four growth scores (score followed by number of free moving Leptospira bacteria per microscopic field at100× magnification): score 0, 0; score 1, 1–5; score 2, 6–10; score 3, 11–50; score 4, >50. The GIT titre of the serum sample was defined as the log_2_ value of the reciprocal of the highest dilution that showed a growth score of ≤2.

### 2.7. Body Temperature

The body temperature was measured prior to challenge (days −3 and 0), and then daily until 28 days after challenge. In studies 1 and 2, subcutaneous transponders, and in studies 3 and 4, rectal thermometers were used to measure the body temperatures, respectively.

### 2.8. Clinical Signs

All dogs were observed for any abnormal clinical signs before challenge (days −3 and 0) and twice daily from day 1 after challenge until 28 days after challenge. Where the observed clinical signs could be confirmed by means of a concurrent abnormal laboratory finding (see Experimental Design), an individual clinical score was recorded as follows; a clinical score of 1 was given to conjunctival suffusion (redness), pale conjunctival or oral mucosa, reduced appetite, vomiting, diarrhoea, and jaundice; a clinical score of 2 was given to reduced skin turgor (indicative of dehydration), no appetite, weakness, slow/lethargic or stiff gait, arched back, extensive petechiae, and wasting. A total clinical score was recorded, being the sum of scores of all individual clinical signs per observation per dog. A total clinical score of 150 was given to each dog that was euthanised because the humane endpoint was reached. Euthanasia was carried out after adequate sedation.

### 2.9. Culturing of Challenge Organisms from Blood, Urine, Kidney, and Liver

Whole blood sampling was done prior to challenge (day −3) and on days 1, 2, 3, 4, 7, 10, 14, and 21 post-challenge. Aliquots of 0.5 mL were, directly from the syringe, inoculated into 10 mL of EMJH medium containing 200 μg/mL 5-fluoro-uracil (5-FU) and 1% (v:v) rabbit serum negative for antibodies against the four vaccine serogroups of Leptospira. Urine sampling by puncture of the bladder was done prior to challenge (day −3) and on days 3, 7, 14, 21, and 28 after challenge. For this purpose, the dogs were treated with a diuretic (furosemide, 5 mg per kg body weight, given intravenously). One ml of urine was directly inoculated into 10 mL of the EMJH medium, as described above. During necropsy, a piece of 1–2 g from the cortex of one of the kidneys as well as a piece of 1–2 g of the liver were taken for culture. The fragments—taken aseptically—were placed in 10 mL of the EMJH medium as described above and homogenised with an Ultraturrax^®^ homogeniser. A 100-fold dilution of each kidney or liver homogenate in the same EMJH medium was used for culturing. Cultures of blood, urine and kidney and liver tissues were incubated at 29 °C and observed weekly using dark-field microscopy for the presence of typical *Leptospira*-shaped, motile bacteria for a total of at least 8 weeks, before negative cultures were discarded. In each study, the duration of bacteraemia (leptospiraemia) was expressed in the number of days of positive blood cultures, and the duration of urinary shedding was expressed in the number of days of positive urine and kidney cultures.

### 2.10. Rapid Urinalysis

In addition to the urine aliquots used for culturing, at least 1.5 mL of the collected urine per dog was taken for rapid urinalysis. Vet-10 urine strips (Kruuse, Denmark) were used to perform an easy-to-read test of freshly sampled urine, for semiquantitative assessment of the following parameters: density (specific gravity), pH, protein, urobilinogen, bilirubin, nitrite, ketones, glucose, leukocytes, and blood. According to the manufacturers’ instructions, strips were dipped in urine samples and the results were read after 30–60 s by comparison of the colouring reactions with the colour scale.

### 2.11. Post-Mortem Examination

Necropsy was carried out in all cases where the humane endpoint was reached and for all surviving dogs at the end of the study (day 28 post-challenge). Necropsy was performed immediately after euthanasia. Macroscopic examination was done with special attention paid to the lungs, liver, kidneys, and spleen. Histopathological examination was performed with tissue samples (preferably taken from lesions) from liver, kidneys, spleen, and from any organ/tissue with suspected lesions. All tissue samples were processed, and sections were stained with haematoxylin and eosine (HE) for histopathological examination according to standard procedures.

### 2.12. Evaluation of Results and Statistical Analysis

For comparison of results of serology, urinalysis, and post-mortem examination between the treatment groups, a qualitative assessment of data of the groups was done and no inferential statistical analysis was used (i.e., only descriptive statistics were conducted). Group comparisons of lab-confirmed, clinical signs post-challenge (total clinical score per dog) were conducted using the Kruskal-Wallis test for all three groups and, if significant, was followed by pairwise comparisons using the Wilcoxon rank-sum test. Group comparisons of body temperature were conducted for days on which challenge effects were expected or actually observed using the Kruskal-Wallis test for all three groups and, if significant, were followed by pairwise comparisons using the Wilcoxon rank-sum test. This analysis was conducted for the following days post-challenge: study 1, days 2, and 5; study 2, days 2 and 3; study 3, days 2, and 3; study 4, days 1, and 12. Culturing results (number of days with positive blood samples per dog and number of days with positive urine and kidney samples per dog) were analysed using the Kruskal-Wallis test for all three groups and, if significant, were followed by pairwise comparisons using the Wilcoxon rank-sum test. For all parameters, tests were two-sided, using a significance level (alpha) of 5%. The statistical software package SAS V9.4 (SAS Institute Inc. Cary, NC, USA) was used for the inferential statistical analysis.

## 3. Results

### 3.1. Serology

**Figure 1:**
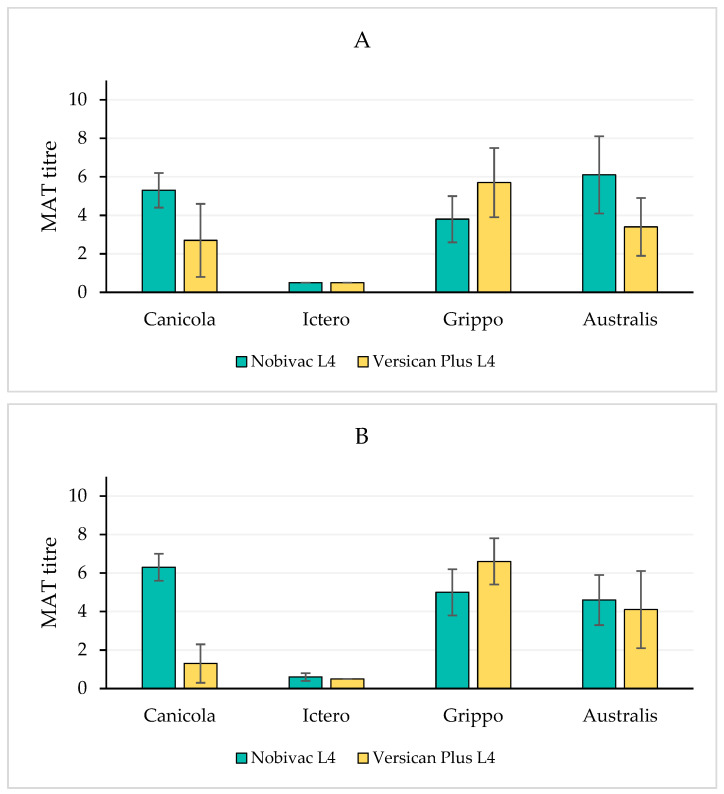

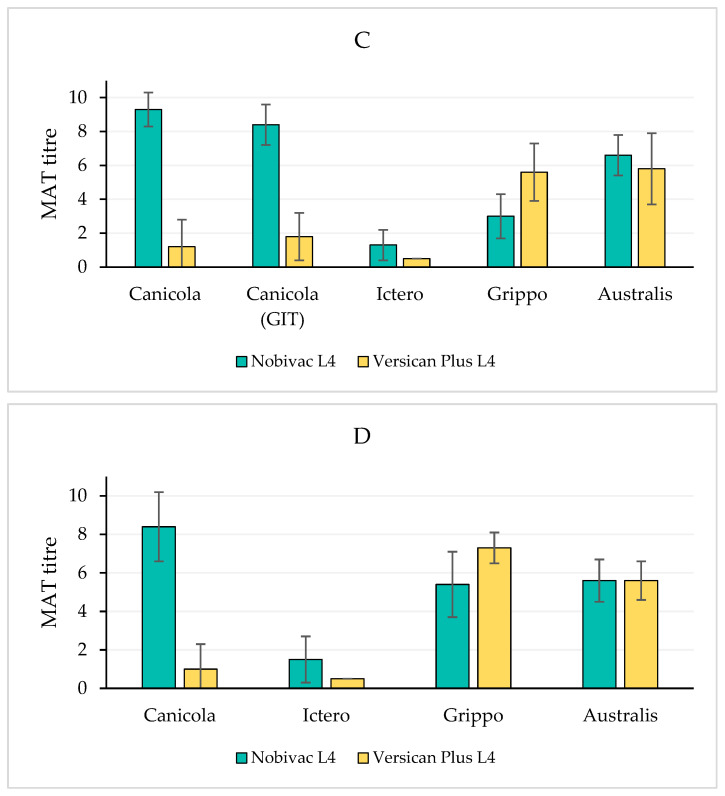
(**A**): Study 1, Grippotyphosa challenge; (**B**): Study 2, Icterohaemorrhagiae challenge; (**C**): Study 3, Canicola challenge; (**D**): Study 4, Australis challenge. Pre-challenge titres of agglutinating serum antibodies against the four vaccine serogroups in studies 1-4 and GIT titres against serogroup Canicola in study 3. MAT and GIT antibody titres three weeks after the second vaccination, expressed in log_2_ values. Bars represent mean values and error bars represent standard deviations of the mean.

In Figure 1A–D, the pre-challenge MAT antibody titres (at three weeks after the second vaccination) against the four vaccine serogroups are shown for the four studies. Whereas, in all studies, titres against Grippotyphosa were approximately two log_2_ units higher in the Versican Plus L4 group; the anti-Canicola titres were higher (up to eight log_2_ units higher) than those in the Nobivac L4 group. It is important to note that in the MAT, a culture of serovar Canicola was used as the antigen, which implies that this MAT antigen was homologous to the corresponding vaccine antigen in the Versican Plus L4 vaccine (serogroup Canicola serovar Canicola) and heterologous to the corresponding vaccine antigen in the Nobivac L4 vaccine (serogroup Canicola serovar Portland-vere). This implies that the relatively low anti-Canicola antibody titres in the Versican Plus group were not the result of the MAT antigen (serovar) used, and thereby, this was not a methodological effect, but it represented a true difference in serological response when compared to the that in the Nobivac L4 group. At the group level, the large difference in titres of agglutinating serum antibodies between the two vaccinated groups was associated with a difference in efficacy between the two vaccinated groups against Canicola, as shown by other efficacy parameters (see the sections: Lab-confirmed clinical signs, Culturing of challenge organisms, and Rapid urinalysis, below). The results of the growth inhibition test for serogroup Canicola, which was only carried out in study 3 (with Canicola challenge), showed the same striking difference as the results of the MAT against serogroup Canicola (see Figure 1C). This implies that the titres of agglutinating antibodies as well as growth inhibiting antibodies were profoundly lower in the group vaccinated with Versican Plus L4. This was correlated with differences in the prevalence of clinical signs, duration of bacteraemia, and prevalence of proteinuria and bilirubinuria, all of which were higher or of a longer duration in the group vaccinated with Versican Plus L4.

Antibody titres against the other two serogroups (Icterohaemorrhagiae and Australis) did not show marked differences between the two vaccinated groups.

### 3.2. Body Temperature

**Figure 2:**
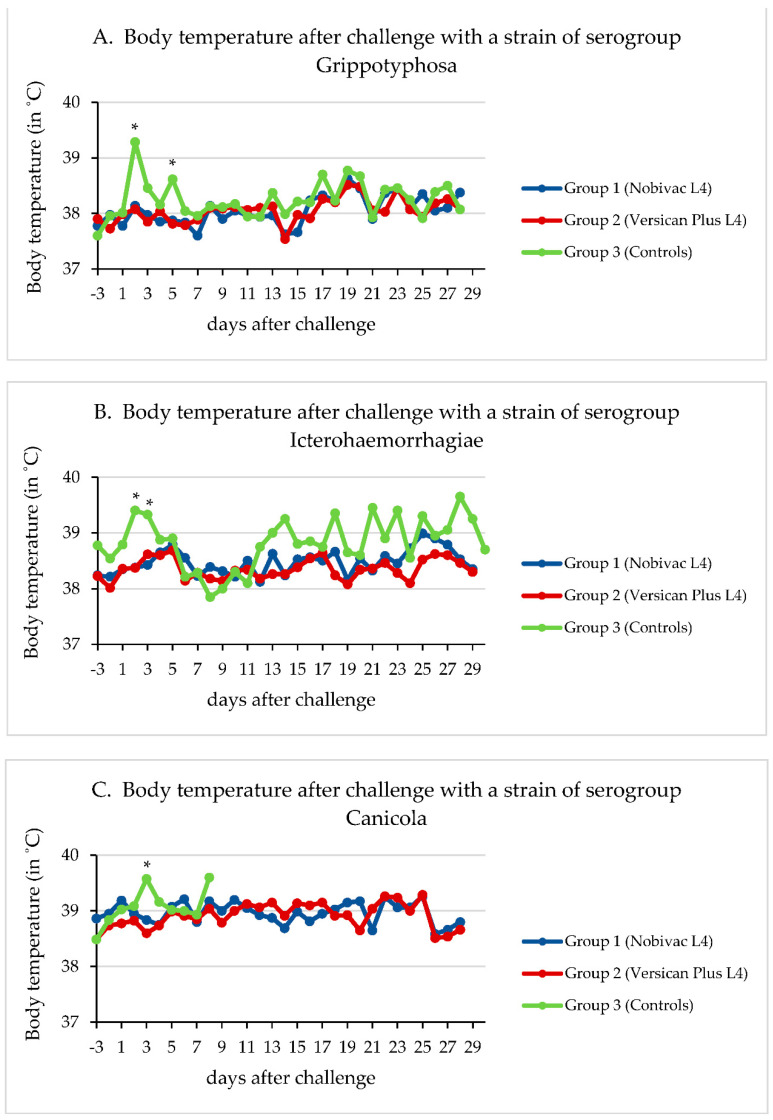

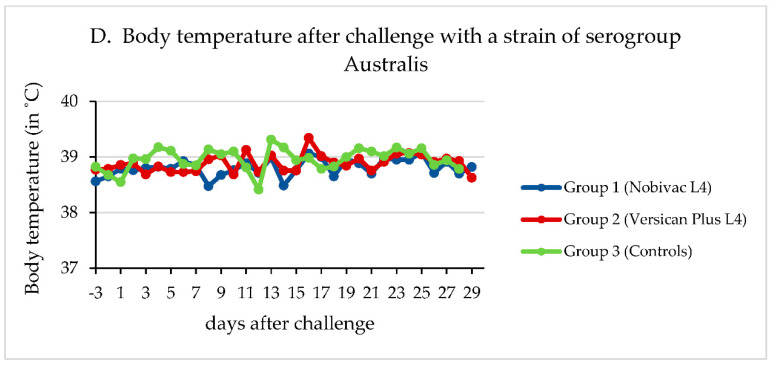
Body temperature after challenge in studies 1–4. The asterisks indicate a significant difference (*p* < 0.05) as follows: (**A**) day post-challenge (dpc) 2: group 1 versus 3: *p* = 0.0054; group 2 versus 3: *p* = 0.0088; dpc 5: group 1 versus 3: *p* = 0.0235; group 2 versus 3: *p* = 0.0481. (**B**) dpc 2: group 1 versus 3: *p* = 0.0395; group 2 versus 3: *p* = 0.0321; dpc 3: group 1 versus 3: *p* = 0.0176; group 2 versus 3: *p* = 0.0320. (**C**) dpc 3: group 1 versus 3: *p* = 0.0202; group 2 versus 3: *p* = 0.0009. (**D**) No statistically significant differences between groups.

In Figure 2A–D, mean body temperatures per group are shown for all four studies, respectively. In these graphs it is demonstrated that the challenges with Grippotyphosa, Icterohaemorrhagiae, and Canicola resulted in one or more evident fever peaks in the control group. Typical biphasic fever patterns were observed in the control groups after the Grippotyphosa and Canicola challenge (as reported in field infections [13]), and multiple peaks were seen after the Icterohaemorrhagiae challenge in two surviving control dogs. In studies 1, 2, and 3, on the days with clear changes in body temperature in the control groups, both vaccinated groups showed a statistically significant difference when compared with that of the control group. In study 4, neither of the two vaccinated groups showed a statistically significant difference with the control group. In this latter study however, two control dogs had a decrease in body temperature of more than 1 °C. One of these dogs, which showed recurrent decreases in temperature, was euthanised on day 12 post-challenge because of severe clinical signs. Importantly, some of the dogs that were euthanised in studies 1 (1 out of 1 euthanised dog), 2 (4 out of 8 euthanised dogs), 3 (1 out of 8 euthanised dogs), and 4 (1 out of 1 euthanised dog), had a decreased temperature on the day of euthanasia (results not shown).

No statistically significant differences in body temperature were detected between the two vaccinated groups for any of the studies.

### 3.3. Lab-Confirmed Clinical Signs

Table 2 shows the average total clinical score of the dogs per group in studies 1–4 based on the clinical signs that were lab-confirmed, i.e., confirmed with positive findings from culturing (blood, urine, kidney, or liver) or urinalysis (proteinuria or bilirubinuria) or histopathology. The most striking finding was that in study 2 (Icterohaemorrhagiae), vaccination with Nobivac L4 induced a statistically significant reduction of the clinical signs when compared to those given a vaccination with Versican Plus L4. In study 1 (Grippotyphosa), although one dog in the Versican group was euthanised and given a clinical score of 150 (laboratory-confirmed clinical signs including reduced appetite, slow, reduced skin turgor, and arched back), the clinical score was statistically significantly reduced in each vaccinated group when compared to that of the control group. In study 4 (Australis), no statistical significance was reached in the comparison of each vaccinated group with the control group. The clinical signs in the two vaccinated groups were very mild and transient (results not shown). In the control group, there were three dogs with conjunctival suffusion and one dog with multiple clinical signs (see Table 2). This dog developed clinical signs from day seven post-challenge onwards, with severe signs on day 12 post-challenge, on which day, the dog was humanely euthanised. The signs of clinical leptospirosis in this dog corresponded with positive culture results from blood, urine, and kidney, proteinuria, bilirubinuria, and post-mortem pathology (see post-mortem results).

### 3.4. Culturing of Challenge Organisms

In Table 3, the duration of bacteraemia (leptospiraemia) and urinary shedding per group in all four studies are shown. In studies 1 (Grippotyphosa), 3 (Canicola), and 4 (Australis), both vaccines resulted in statistically significant reductions of the two parameters when compared to those in the respective control groups. In contrast, in study 2 (Icterohaemorrhagiae), only Nobivac L4 induced a statistically significant reduction in the two parameters. Versican Plus L4 did not show a statistically significant reduction in the two parameters in the Icterohaemorrhagiae study. In addition, in the Icterohaemorrhagiae study, for both parameters, there was a statistically significant difference between the two vaccinated groups. In the Canicola study, there was a statistically significant difference between the two vaccinated groups in the duration of bacteraemia. In the Grippotyphosa and Australis studies, there were no statistically significant differences between the two vaccinated groups.

### 3.5. Rapid Urinalysis

The outcome of all parameters that were measured with the Vet-10 urine strips—on which no statistical comparison was done—showed that the challenge induced positive test results for a urinary protein and bilirubin. In addition, in some of the control dogs in studies 2 and 3, glucose was detected in the urine post-challenge (results not shown). The numbers and percentages of positive dogs and the mean number of positive days per group with proteinuria and bilirubinuria in studies 1–4 are shown in Table 4. For the presence of a protein in the dog urine, the reference range is shown by the manufacturer of the strips, as negative. On the urine strip, the scale for the protein includes the following concentrations: 0 (negative), 0.3, 1.0, and 5.0 g/litre, with the latter three results being presented as positive. In study 1, proteinuria was hardly detected and there were no relevant differences between the groups in occurrence or duration of proteinuria. In studies 2, 3, and 4, the prevalence and duration of proteinuria were consistently higher in group 2 (Versican Plus L4) than they were in group 1 (Nobivac L4). For the presence of bilirubin in the dog urine, the reference range is shown by the manufacturer of the strips, as negative. On the urine strip the scale for bilirubin includes the following concentrations: 0 (negative), 10, 20, and 40 mg per litre, the latter four results being presented as positive. Bilirubinuria was only evaluated in studies 1, 3, and 4 because in study 2, prior to the challenge, bilirubinuria was detected in too many dogs for proper evaluation of the treatment effects. In studies 1, 3, and 4 the prevalence and duration of bilirubinuria were consistently higher in group 2 (Versican Plus L4) than they were in group 1 Nobivac L4). In studies 2 and 3, the low numbers of positive dogs in the respective control groups were an under-representation because the control dogs were euthanised 4–7 days after the challenge, while bilirubinuria, throughout the studies, was mainly detected between days 7 and 28. This implies that a significant number of control dogs would have developed bilirubinuria, if they had survived beyond days 4–7 post-challenge.

The other urine parameters that were examined (see the Materials and methods) did not show any clear abnormality nor any difference between the three groups post-challenge (results not shown).

### 3.6. Post-Mortem Results

No inferential statistics were conducted on the post-mortem results and, therefore, they are not shown in the Tables or Figures.

Predominant macroscopic abnormalities, throughout the four studies (control groups), were swollen kidneys and/or multiple haemorrhages on the surface and in the cortex of the kidneys. The euthanised dog in group 2 of study 1 (Versican Plus L4 vaccine) also had swollen kidneys. In studies 2 and 3, in addition to this, lungs with multiple haemorrhages were observed.

After necropsy, histopathological examinations in studies 1, 2, and 3 demonstrated the presence of interstitial nephritis in 100% of the control dogs. In studies 2, 3, and 4, also, alveolar oedema in the lungs and/or haemorrhages and/or intravascular coagulation in several organs were demonstrated.

The only vaccinated group with a high percentage of dogs with interstitial nephritis (57%) was the Versican Plus L4-vaccinated group in study 2 (Icterohaemorrhagiae), compared with 25% in the Nobivac L4-vaccinated group. In the Versican Plus L4-vaccinated group, the two euthanised dogs had interstitial nephritis as well as extensive lung pathologies. In study 4 (Australis), none of the vaccinated dogs showed histopathological lesions consistent with a detected *Leptospira* infection, whereas two control dogs did. In the control dog that was euthanised because of it showing severe clinical signs (see Lab-confirmed clinical signs), glomerular and interstitial nephritis and severe intrapulmonary haemorrhages and oedema were demonstrated. In the other control dog (with conjunctival suffusion and positive blood and urine culture results), a macroscopic examination showed red areas in the right lung, after which a microscopic examination demonstrated multifocal interstitial mononuclear cell infiltration implying the presence of pneumonitis. The pathology of both dogs was attributed to the Australis (serovar Bratislava) challenge, since both dogs had convincing evidence of infection from clinical as well as laboratory findings, and because renal as well as pulmonary pathology are associated with canine leptospirosis.

For an overall comparative summary of the three main efficacy parameters, see Table 5. This summarises the level of protection against infection, urinary shedding, and clinical signs seen in both vaccine groups, and whether or not any differences were statistically significant.

## 4. Discussion

In the present studies, the protective effects of the two tetravalent vaccines authorised for use in Europe, Nobivac L4 and Versican Plus L4, were compared in two ways; each vaccinated group was compared with the control group and the two vaccinated groups were compared with each other. Versican Plus L4 vaccine was regarded as a “positive control” for protection from the clinical signs of leptospirosis because in a prior publication, the absence of clinical signs in all vaccinates in each challenge study was described [8]. In the latter studies, a challenge was performed four weeks after the second vaccination, whereas in the present studies, for practical reasons, all groups were challenged at three weeks after the second vaccination. In previously published efficacy studies with Nobivac L4 [7], the focus has been on protection from the infection and urinary shedding, rather than the clinical signs, since the latter develop only when the numbers of leptospires in the blood and tissues reach a critical level and trigger lesions due to the action of undefined leptospiral toxin(s) or toxic cellular components [1]. For this reason, the presence of clinical signs in the studies with Nobivac L4 was not used as a primary measure of efficacy and, consequently, the protective effects against clinical signs are not an explicit part of the efficacy claims of this vaccine—although, of course clinical protection is assumed. For the present studies, in order to achieve a higher prevalence of clinical signs in the non-vaccinated control groups in comparison with the previous studies [7], a more severe infection model was used. This involved the hamster passage of the Leptospira challenge strains prior to the dog challenge for all the challenge studies except for the strain of serogroup Canicola, which was sufficiently virulent without the hamster passage. Also, an intranasal administration was used as an additional challenge route, next to the intraperitoneal and conjunctival routes used in previous studies, in order to expose the dogs to a higher dose of leptospires via the mucosal system, which better reflects the natural infection route.

In the present studies, serovars Copenhageni and Bananal/Liangguang were used for the challenge, whereas serovars Icterohaemorrhagiae and Grippotyphosa were used by Wilson et al. [8]. This implies that the efficacy results of the present serogroup Icterohaemorrhagiae and Grippotyphosa studies cannot, therefore, be compared with those of the corresponding studies described by Wilson et al. [8], without taking into account these differences in challenge serovars. The results of the serogroup Canicola and Australis studies, however, may be more comparable between the present studies and the corresponding studies by Wilson et al. [8] since the same serovars were used for challenge. The outcome of the present studies was that the proportion of dogs with clinical signs in the non-vaccinated control groups was 100%, except for the serogroup Australis challenge where only 50% of the control dogs had clinical signs. In addition, the proportion of control dogs with positive urine or kidney cultures in the present Australis (serovar Bratislava) study was 75%. Since these proportions of positive control dogs are substantially higher than they were in the previous studies [7], this confirms the increased virulence of the three strains as a result of the hamster passage. A statistical analysis demonstrated significant differences in the clinical scores between each vaccinated group and the control group in the Grippotyphosa, Icterohaemorrhagiae, and Canicola challenge studies. In the Icterohaemorrhagiae study, the difference in clinical scores between the two vaccinated groups was also statistically significant.

The results of the protection from the clinical signs in the groups vaccinated with Versican Plus L4 were, in part, different from those described in previous studies [8]. Versican Plus L4 resulted in a reduction, but not a prevention, of clinical signs after the challenge with a strain of serogroup Grippotyphosa. With both vaccines, at least a statistically significant reduction of the duration of infection and urinary shedding was achieved for serogroups Grippotyphosa, Canicola, and Australis. For serogroup Icterohaemorrhagiae (serovar Copenhageni), however, Versican Plus did not induce a statistically significant reduction with these two parameters. When focusing on the two studies in which the same challenge serovars (Canicola and Bratislava) were used as were used in the studies by Wilson et al. [8], it was concluded that the efficacy results with Versican Plus L4 in the present Canicola study were different from those in the Canicola study by Wilson et al. [8]. In the latter study, all the dogs in the Versican Plus L4 group were completely protected (implying prevention) from infection, urinary shedding, and clinical signs, whereas in the present study no prevention of infection or urinary shedding or clinical signs was seen in this group. This was associated with a relatively high prevalence of proteinuria and bilirubinuria and relatively low pre-challenge antibody titres against Canicola when compared with the results of the Nobivac L4 group, as demonstrated by the MAT as well as the growth inhibition test. Relatively low MAT titres against Canicola in the Versican Plus L4 group after two vaccinations were measured throughout the four present studies using a MAT antigen of the same serovar as was used in the vaccine antigen in Versican Plus L4, i.e., serovar Canicola. Because of the limited availability of dog sera and in order to focus on the Canicola study, the growth inhibition test was only performed with the sera of the Canicola study. For this study, it was demonstrated that titres of agglutinating as well as growth inhibiting serum antibodies were very low in the group vaccinated with Versican Plus L4. Two batches of each vaccine were used; one batch in studies 1 and 2, and the other batch in studies 3 and 4, and the large difference in MAT titres against Canicola throughout the four studies and between the two vaccinated groups was observed with both batches of each product.

To the authors’ knowledge, a rapid urinalysis was used for the first time to detect the gross negative effects of a challenge on renal or hepatic function. The rapid urinalysis results showed that, as a result of successful infection, protein and/or bilirubin were detected in the urine, which is in accordance with laboratory findings in clinical infections [15,16]. In studies 2 and 3, the low numbers of dogs with proteinuria in the respective control groups are an under-representation because control dogs were euthanised 4–7 days after the challenge, while proteinuria, throughout the groups, was mainly detected between days seven and 28. Although it is known that in adult, clinically normal male dogs, especially those with high urine specific gravity, a small amount of bilirubin is typically present in the urine [17], in three of these studies, the Leptospira challenge resulted in bilirubinuria that was not observed prior to the challenge. The highest bilirubin concentrations in the urine post-challenge were observed in the Grippotyphosa study.

To the authors’ knowledge this is also the first study in which experimental infection of dogs with a strain of serovar Bratislava has resulted in such severe clinical signs that the humane endpoint was reached in one of the non-vaccinated control dogs that, therefore, had to be humanely euthanised. This happened 12 days after the challenge with a relatively low dose of the challenge bacteria (in total 5.8 mL containing 3.5 × 10^7^ cells/mL). This was in contrast with a study by Greenlee et al. [18] in which the challenge of dogs on three consecutive days with a strain of serovar Bratislava did not result in any leptospiraemia, urinary shedding of challenge organisms, or clinical disease. Although the majority of the control dogs in the present study had mild or no clinical signs, this study has clearly demonstrated the potential role of serovar Bratislava in the development of severe clinical leptospirosis in dogs, which is in accordance with a recently published report of some clinical cases [19].

## 5. Conclusions

It was concluded that, when compared to vaccination with Versican Plus L4, vaccination with Nobivac L4 resulted in generally better control of the leptospirosis disease parameters after a challenge including the complete prevention of clinical signs following a Grippotyphosa and Icterohaemorrhagiae challenge. In contrast, the vaccination with Versican Plus L4 only prevented infection by Australis and shedding by Grippotyphosa and Australis, but it did not result in any statistically significant reduction on either infection or shedding following the Icterohaemorrhagiae challenge.

## Figures and Tables

**Table 1:** Treatment schedule of studies 1–4 ^1^: grouping, dosage and administration.

Group	n ^2^	Vaccine ^3^	1st Vaccination		2nd Vaccination		Challenge	
			Flank, Left		Flank, Right			
			Age (wks)	Dose	Age (wks)	Dose	Age (wks)	Dose
1	8	Nobivac L4	6	1 mL, s.c. ^4^	10	1 mL, s.c.	13	i.p. ^4^: 4.5 mL, i.n. ^4^: 0.4 mL each nostril, conj. ^4^: 0.25 mL each eye
2	8	Versican Plus L4		1 mL, s.c.		1 mL, s.c.		
3	8	none		none		none		

^1^ Study 1, challenge with strain of serogroup Grippotyphosa; study 2, challenge with strain of serogroup Icterohaemorrhagiae; study 3, challenge with strain of serogroup Canicola; study 4, challenge with strain of serogroup Australis. ^2^ In the following studies and groups, there were 7 dogs due to the exclusion of one dog due to ill health (e.g., growth retardation) prior to the challenge: group 3 in study 1; group 2 in study 2; group 2 in study 4. ^3^ In studies 1 and 2, Nobivac^®^ L4 batch 172668.1 and Versican^®^ Plus L4 lot 316222A02 were used; in studies 3 and 4, Nobivac^®^ L4 batch 172714 and Versican^®^ Plus L4 lot 696223A01 were used. ^4^ s.c., subcutaneous; i.p., intraperitoneal; i.n., intranasal; conj., conjunctival instillation.

**Table 2:** Scores of laboratory-confirmed clinical signs of leptospirosis in studies 1–4.

Study (Challenge Serogroup)	Group (Vaccine)	Clinical Score Expressed in Mean per Group (Number of Dogs with One or More Clinical Signs)	Clinical Signs Observed per Study (One or More of the Following)	Comparison of Clinical Scores: Differences between Groups ^1^
1 (Grippotyphosa)	1 (Nobivac L4)	0.0 (0/8)	Reduced appetite, slow/stiff gait, weakness, bloody diarrhoea, pale mucosa, reduced skin turgor, arched back	1–3: 0.0005 * 2–3: 0.0123 *^,2^ 1–2: 0.3816
	2 (Versican Plus L4)	18.8 (1/8) ^2^		
	3 (control)	8.6 (7/7)		
2 (Icterohaem-orrhagiae)	1	0.0 (0/8)	Reduced appetite, slow/stiff gait, weakness, pale mucosa, reduced skin turgor, dyspnoea	1–3: 0.0003 * 2–3: 0.0272 * 1–2: 0.0209 *
	2	43.1 (4/7)		
	3	118.0 (8/8)		
3 (Canicola)	1	0.8 (2/8)	Slow/stiff gait, weakness, bloody diarrhoea, pale mucosa, conjunctival suffusion (=red conjunctiva), arched back	1–3: 0.0003 * 2–3: 0.0004 * 1–2: 0.1473
	2	3.1 (5/8)		
	3	150.0 (8/8)		
4 (Australis)	1	0.1 (1/8)	No appetite, slow/stiff gait, weakness, bloody diarrhoea, pale mucosa, conjunctival suffusion, reduced skin turgor, arched back	NS
	2	0.3 (2/7)		
	3	19.6 (4/8)		

^1^*p*-values are given for the comparisons of clinical scores of group 1 (Nobivac L4) with group 3 (control group), group 2 (Versican Plus L4) with group 3, and group 1 with group 2; when statistically significant, *p*-values are followed by an asterisk; NS, no statistically significant differences among three groups (Kruskal-Wallis *p*-value = 0.1722). ^2^ Relatively high group mean value was caused by clinical score of 150 of one dog (that was euthanised); however, statistical analysis showed that group 2 had significantly lower clinical scores than group 3 did.

**Table 3:** Duration of bacteraemia and urinary shedding.

Study (Challenge Serogroup)	Group (Vaccine)	Group Size	Days of Positive Blood Cultures (Mean) ^1^	Differences between Groups (Blood Cultures) ^2^	Days of Positive Urine& Kidney Cultures (Mean) ^1^	Differences between Groups (Urine and Kidney Cultures) ^2^
1 (Grippo- typhosa)	1 (Nobivac L4)	8	0.3	1–3: 0.0005 * 2–3: 0.0005 * 1–2: 1.0000	0.0	1–3: 0.0005 * 2–3: 0.0005 * 1–2: 1.0000
	2 (Versican Plus L4)	8	0.3		0.0	
	3 (control)	7	4.9		2.3	
2 (Icterohaem-orrhagiae)	1	8	2.8	1–3: 0.0017 * 2–3: 0.0864 1–2: 0.0181 *	1.5	1–3: 0.0013 * 2–3: 0.1423 1–2: 0.0326 *
	2	7	5.6		4.1	
	3	8	7.3		5.0	
3 (Canicola)	1	8	0.3	1–3: 0.0002 * 2–3: 0.0004 * 1–2: 0.0345 *	0.0	1–3: 0.0004 * 2–3: 0.0018 * 1–2: 0.0764
	2	8	2.5		0.8	
	3	8	8.0		4.0	
4 (Australis)	1	8	0.0	1–3: 0.0044 * 2–3: 0.0071 * 1–2: 1.0000	0.0	1–3: 0.0045 * 2–3: 0.0073 * 1–2: 1.0000
	2	7	0.0		0.0	
	3	8	3.1		1.5	

^1^ In total 9 blood samples were taken for culturing (on days −3, 1, 2, 3, 4, 7, 10, 14, and 21 post-challenge), and in total 6 urine samples were taken for culturing (on days −3, 3, 7, 14, 21, and 28 post-challenge). ^2^ *p*-values are given for the comparisons of group 1 (Nobivac L4) with group 3 (control group), group 2 (Versican Plus L4) with group 3, and group 1 with group 2; when statistically significant, *p*-values are followed by an asterisk.

**Table 4:** Prevalence and duration of proteinuria or bilirubinuria.

Study (Challenge Serogroup)	Group (Vaccine)	Proteinuria		Bilirubinuria	
		Number of Positive Dogs (Percentage)	Mean Number of Positive Days ^1^	Number of Positive Dogs (Percentage)	Mean Number of Positive Days ^1^
1 (Grippotyphosa)	1 (Nobivac L4)	2/8 (25%)	0.25	2/8 (25%)	0.25
	2 (Versican Plus L4)	1/8 (13%)	0.13	7/8 (88%)	0.88
	3 (control)	1/7 (14%)	0.14	6/7 (86%)	1.14
2 (Icterohaem- orrhagiae)	1	3/8 (38%)	0.38	Not valid ^3^	
	2	5/7 (71%)	0.71		
	3	0/8 (0%) ^2^	0.00		
3 (Canicola)	1	2/8 (25%)	0.38	2/8 (25%)	0.25
	2	5/8 (63%)	0.75	5/8 (63%)	0.88
	3	3/8 (38%) ^2^	0.63	5/8 (63%)	0.88
4 (Australis)	1	2/8 (25%)	0.25	4/8 (50%)	0.50
	2	4/7 (57%)	0.71	6/7 (86%)	1.29
	3	5/8 (63%)	0.75	4/8 (50%)	0.75

^1^ In total, 6 urine samples were taken for rapid urinalysis (days −3, 3, 7, 14, 21, and 28 post-challenge). ^2^ In studies 2 and 3, low number of dogs with proteinuria in control group is an under-representation because most control dogs were euthanised 4–7 days after challenge, while proteinuria, throughout the groups, was mainly detected between days 7 and 28. ^3^ Bilirubinuria was detected before challenge in seven dogs, including three control dogs.

**Table 5:** Summary of comparative efficacy of the two vaccines in the present four studies.

Efficacy Against	Challenge Serogroup ^1^	Nobivac L4 ^2^	Versican Plus L4 ^2^	Outcome Statistical Test ^3^
Infection (leptospiraemia)	Grippotyphosa	Reduction	Reduction	Not significant
	Icterohaemorrhagiae	Reduction	Not significant ^1^	Nobivac L4
	Canicola	Reduction	Reduction	Nobivac L4
	Australis	Prevention	Prevention	Not significant
				
Urinary shedding (leptospiruria)	Grippotyphosa	Prevention	Prevention	Not significant
	Icterohaemorrhagiae	Reduction	Not significant	Nobivac L4
	Canicola	Prevention	Reduction	Not significant
	Australis	Prevention	Prevention	Not significant
				
Clinical score (laboratory confirmed)	Grippotyphosa	Prevention	Reduction	Not significant
	Icterohaemorrhagiae	Prevention	Reduction	Nobivac L4
	Canicola	Reduction	Reduction	Not significant
	Australis	Not significant	Not significant	Not significant

^1^ In the present studies, serovar Copenhageni of serogroup Icterohaemorrhagiae and serovar Bananal/Liangguang of serogroup Grippotyphosa were used for challenge, whereas serovars Icterohaemorrhagiae and Grippotyphosa were used in the previous work by Wilson et al. [8]. ^2^ Comparison of Nobivac L4 group with control group or comparison of Versican Plus L4 group with control group. ^3^ Comparison of Nobivac L4 group with Versican Plus L4 group; in case of statistically significant difference, the vaccine with the most significant protection is indicated.

## Data Availability

Not applicable.
